# What Is a Cell?

**Published:** 1891-09

**Authors:** 


### WHAT IS A CELL?

The word "cell" is now in common use in all physiological and medical discussions. Yet thousands, if called upon to give a definition of it, would find themselves nonplussed or greatly puzzled. Some of the commonest words, as love, religion, truth, are almost indefinable, and the difficulties of definition generally increase with simplicity and frequency of use. Let us see if we can tear this little word to pieces, for the learned word analysis means, in plain speech, only tearing to pieces. First as to its parentage. In the Latin the little word *cella* means a small cavity, and it is related to *celare*, to conceal. This is the parent of the word cell. Webster says the biological meaning of the word is "One of the minute elementary structures of which the greater part of the various tissues and organs of animals and plants are composed." It is microscopical in size; seen through a microscope it appears like a little sac, spheroidal (that is, like a sphere, but not quite spherical, roundish), in shape, a chemical compound of six elements, viz., oxygen, hydrogen, nitrogen, carbon, sulphur and phosphorus.

Let us glance for a moment at these six constituents of a cell. All matter exists, as a general rule, as a solid, liquid or gas. Oxygen, hydrogen and nitrogen are known to us as gases, and carbon is ever a solid. Five of these elements have a double or changeable character; thus, phosphorus may exist either as a yellow and most inflammable solid, or as a red and singularly incombustible one; carbon, as the very different substances, plumbago, charcoal, or the diamond; oxygen, as simple oxygen or as ozone. Sulphur is known in three distinct characters: while hydrogen is suspected of being the parent of all the so-called elements, itself the sole, final, real element. And then, nitrogen, the really most wonderful of them all, is the basis of all life (so much so that the phrase, "There is no life without nitrogen," has long since become a recognized axiom to which no exception is known, either in the vegetable or the animal world), is the essential ingredient of all our explosives, and is especially curious as so fickle a gas that in all its chemical unions it flies away on the smallest provocation, the very individualist and anarchist of chemistry.

The cell is always colloid. All solid matter is either crystalloid or colloid. The crystalloid is a firm solid. The colloid is a jelly. The crystalloid always grows from accretion or addition from without. The colloid can grow from increase of its constituent parts. The crystalloid is generally dissolvable in water. The colloid is rarely soluble in water. The colloid is the form of all living organic matter.

The word colloid is from the Greek, *Kolla*, glue, and *oidos*, like unto, so that colloid is that which is like unto glue, gelatine, jelly.

It has four parts. First, an outside envelope called the wall, though this is sometimes wanting; second, a fluid called the protoplasmic water; third, the kernel or interior cell within the cell, called the nucleus; fourth, the very inmost marrow and root of the cell within the nucleus, called the nucleolus, the little or lesser nucleus. We trust that our readers will not quarrel with us for this torrent of technicalities. As John G. Saxe once said, "We come to hard words when we meddle with Greek." But they are unavoidable in even the briefest consideration of the subject.—*The Healthy Home.*

